# Y chromosome is moving out of sex determination shadow

**DOI:** 10.1186/s13578-021-00741-y

**Published:** 2022-01-04

**Authors:** Raheleh Heydari, Zohreh Jangravi, Samaneh Maleknia, Mehrshad Seresht-Ahmadi, Zahra Bahari, Ghasem Hosseini Salekdeh, Anna Meyfour

**Affiliations:** 1 Basic and Molecular Epidemiology of Gastrointestinal Disorders Research Center, Research Institute for Gastroenterology and Liver Diseases, Shahid Beheshti University of Medical Sciences, Tehran, Iran; 2 Department of Biochemistry, Faculty of Medicine, Baqiyatallah University of Medical Sciences, Tehran, Iran; 3 Department of Basic Science and Advanced Technologies in Biology, University of Science and Culture, Tehran, Iran; 4 Department of Physiology and Medical Physics, Faculty of Medicine, Baqiyatallah University of Medical Sciences, Tehran, Iran; 5 Department of Molecular Sciences, Macquarie University, Macquarie Park, NSW Australia; 6 Department of Stem Cells and Developmental Biology, Cell Science Research Center, Royan Institute for Stem Cell Biology and Technology, ACECR, Tehran, Iran

**Keywords:** Y chromosome, Sex differences, Cancer, Diseases, Male infertility, Inflammation, Neurodegenerative disorders, Germ cell tumors, Prostate cancer, Hepatocellular carcinoma

## Abstract

Although sex hormones play a key role in sex differences in susceptibility, severity, outcomes, and response to therapy of different diseases, sex chromosomes are also increasingly recognized as an important factor. Studies demonstrated that the Y chromosome is not a ‘genetic wasteland’ and can be a useful genetic marker for interpreting various male-specific physiological and pathophysiological characteristics. Y chromosome harbors male‑specific genes, which either solely or in cooperation with their X-counterpart, and independent or in conjunction with sex hormones have a considerable impact on basic physiology and disease mechanisms in most or all tissues development. Furthermore, loss of Y chromosome and/or aberrant expression of Y chromosome genes cause sex differences in disease mechanisms. With the launch of the human proteome project (HPP), the association of Y chromosome proteins with pathological conditions has been increasingly explored. In this review, the involvement of Y chromosome genes in male-specific diseases such as prostate cancer and the cases that are more prevalent in men, such as cardiovascular disease, neurological disease, and cancers, has been highlighted. Understanding the molecular mechanisms underlying Y chromosome-related diseases can have a significant impact on the prevention, diagnosis, and treatment of diseases.

## Background

**Fig. 1:**
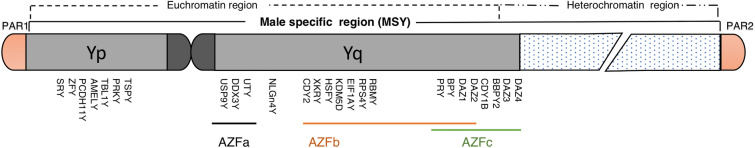
Schematic representation of human Y chromosome. The location of the azoospermia factor (AZF) regions (a–c) and harboring genes have been shown on Yq. The location of some other genes on Yp has also been indicated

**Fig. 2:**
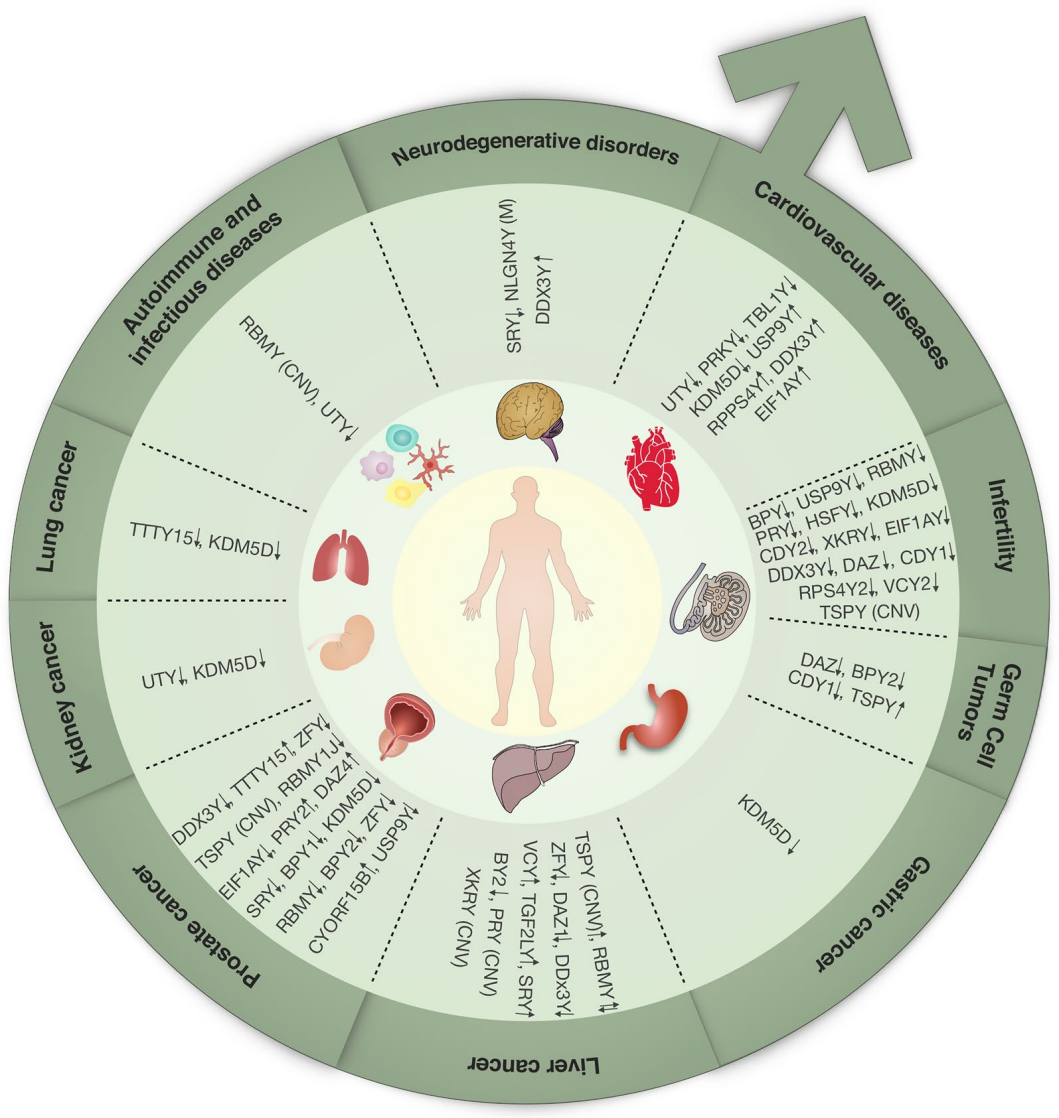
Overview of Y chromosome genes in different diseases. The map shows Y chromosome genes whose expression and/or function have been confirmed in different diseases. CNV, Copy number variation; M, mutation; , down-regulation; , up-regulation

The human Y chromosome is a haploid male-specific chromosome. It consists of about 60 million base pairs and approximately compromises 2% of the human genome [1]. From the evolution point of view, X and Y chromosomes started to evolve from a pair of ancestral autosomes about 25 million years ago [2]. About 95% of the Y chromosome is composed of the male-specific region of the Y chromosome (MSY), and the other 5% is two pseudoautosomal regions (PAR1 and PAR2) in two ends of this chromosome (Fig. 1). PAR1 and PAR2 with less than 3 Mb in length are the only regions that have maintained the ability to recombine with their X counterparts; therefore, MSY escapes meiotic recombination [3]. Based on evolutionary origin, euchromatic sequences of MSY are divided into three different classes: X-degenerate, X-transposed, and ampliconic sequences. X-degenerate sequences are single copy and broadly expressed genes which were evolved from ancestral autosomes to generate sex chromosomes. Their X homologs excessively escape X chromosome inactivation, thus researchers classified them as dose-sensitive and haplolethal genes. The X-transposed region is a result of a recent X-to-Y transposition that has preserved 99% similarity to their X chromosome sequences. Ampliconic sequences, as the largest part of the MSY, encode nine gene families which were acquired from diverse sources and then have undergone amplification [1]. Although the number of MSY genes and their X-homologs is small, they have remained conserved in the human genome over time due to their crucial functions [1]. The role of MSY genes in important cellular processes such as transcription regulation, translation, and protein stability in males is vital not only in sex determination but also in sex-dependent organ development [3]. It has been reported that testis, brain, heart, and kidney developments are associated with MSY genes expression [4, 5]. Despite extensive studies on the effect of these genes on the development pathways, some MSY genes have remained as missing proteins with no experimental protein evidence due to highly transient and spatio-temporal restricted expression patterns. For example, TBL1Y is a crucial protein in cardiac differentiation whose expression was first detected during embryonic stem cell differentiation into cardiomyocytes [6]. Furthermore, there are numerous reports on the direct linkage of MSY genes malfunction with several male-specific disorders, as well as gender differences in prevalence and severity of diseases [7–9]. The occurrence of these differences has been observed in both genetic and non-genetic disorders; for example, autism is four times more prevalent in males than females [7]. Although sex-related circulating hormones have been proposed as one of the causes of these differences, Y chromosome genes, with the cooperation of these hormones or independently, may be responsible for above mentioned sexual disparities [8, 10, 11]. In this article, the role of Y chromosome in male-specific diseases (male infertility and prostate cancer (PC), and the ones which primarily affect men such as cardiovascular diseases, inflammatory diseases, and various types of cancers has been reviewed (Fig. 2).

## Y chromosome in male infertility

Infertility affects an estimated 15% of couples worldwide and male factors are responsible for about 40% of infertile cases [12]. It has been estimated that more than 2.5% of the male infertility cases occur due to chromosomal abnormalities, among which 1.14% are referred to as sex chromosomal abnormalities [13], such as the structural chromosomal abnormalities of the long arm of the Y chromosome (Yq) [14].

The role of the Y chromosome in male infertility has been extensively studied (for review, see refs [3, 15]). Current knowledge of the function of MSY genes in spermatogenesis is mainly based on the reported microdeletions in the Y chromosome of infertile men. The azoospermia factor (AZF) region which is located in Yq, harbors three subregions (AZFa, AZFb, and AZFc) (Fig. 1) involved in sperm development and function [1, 16]. Almost 25–55% of males with different testicular pathologies such as sperm maturation arrest, sertoli cell-only syndrome (SCOS), hypospermatogenesis, and 5–25% of males with severe oligozoospermia or azoospermia show microdeletions in AZF regions [3]. In addition to AZF complete deletion, the association of partial AZFc deletion such as b1/b3, b2/b3, and gr/gr with male infertility has also been reported [3]. AZFc and AZFb deletions transpired in about 60% and 6–10% of azoospermic patients, respectively, although these statistics can vary in distinct human populations. Recently, the association of AZFb deletions with variable testicular pathologies including meiotic arrest, cryptozoospermia, severe oligozoospermia, or oligoasthenoteratozoospermia has been comprehensively reviewed by Vogt et al. (see [17]).

Deletion in the *DDX3Y* and *USP9Y* genes located in the AZFa region is highly associated with the SCOS phenotype [18]. RNA-binding motif protein Y chromosome (RBMY) and PTPN13-like protein Y-linked (PRY) are the main players during spermatogenesis. RBMY is expressed in spermatogonia, spermatocytes, and round spermatids, indicating its important function as a testis-specific splicing factor in germ cell development. Furthermore, it has been reported that complete meiotic arrest is caused by deletions in *RBMY* and *PRY* genes [16]. *PRY* encodes a tyrosine phosphatase protein which is involved in the apoptosis process required to remove sperm cells carrying chromosomal abnormalities [16, 19]. Alteration in the expression of *HSFY*, *KDM5D/SMCY*, *CDY2*, *XKRY*, *EIF1AY,* and *RPS4Y2*, which are located in the AZFb region, may lead to deteriorated spermatogenesis [16, 20, 21].

*DAZ*, *CDY1*, *BPY2,* and *PRY* are some genes located in AZFc which can be directly related to the incidence of oligozoospermia and azoospermia [22]. *DAZ* genes encode RNA-binding proteins which are crucial in all stages of spermatogenesis. *DAZ* expression induces the differentiation of pluripotent stem cells (PSC) toward primordial germ cell-like cells and promotes their maturation [23, 24].

CDY1 consists of a chromodomain and a histone acetyltransferase catalytic domain, whose expression is required for histone-protamine replacement in late spermatid nuclei [25]. VCY2 is a highly positive charged protein that is highly expressed in spermatogonia, spermatocytes, and round spermatids. It interacts with ubiquitin-protein ligase E3A, whose expression has been confirmed in ejaculated human spermatozoa, showing their possible positive effect on the preservation of sperm fertility [26, 27]. Differentially expression of some genes on human Y-chromosome such as *HSFY*, *BPY* has been reported in maturation arrest (MA) patients compared to the normal group [28]. Ahmadi Rastegar et al. introduced an isoform level signature of MSY genes to discriminate among MA, SCOS, and normal testicular tissues, which can be considered as a diagnostic marker for the presence of mature sperm cells in candidate azoospermia men for surgery [28].

In addition to deletion and partial deletions, copy number variations (CNV) of Y chromosome genes can also cause spermatogenesis failure and male infertility. A high-throughput ligation-dependent probe amplification (HLPA) assay was designed to analyze CNVs in the 115 genomic loci covering the Y chromosome. The findings revealed that men with low sperm concentration (LSC) have lower copy numbers for heterochromatic sequences compared with the normal semen group [29]. Chen et al. for the first time, reported that ultra-low relative copy number (RCN) type and low RCN type in Yq12 are more related to male infertility [29]. In contrast, the relation between the additional copy numbers of *TSPY* and spermatogenic failure has also been observed [30].

Chromosomal microarray analysis (CMA) of Y-linked CNVs showed that both CNV size and the involvement of spermatogenesis-related genes determine the clinically relevant CNVs in infertile men [31]. Insufficient copy numbers of the *RBMY* gene can result in asthenozoospermia [32]. CNVs of *DAZ*, *CDY1*, and *BPY2* are correlated with decreased total motile sperm count and lead to azoospermia and moderate/severe oligozoospermia phenotypes [31, 33]. However, screening and detection of mosaic loss of chromosome Y (mLOY) by semi‐quantitative multiplex polymerase chain reaction (PCR) and droplet digital PCR showed the infrequency of leukocyte mLOY in young men with spermatogenic failure [34].

## Y chromosome in neurodevelopmental and neurodegenerative disorders

About 7 million people worldwide die each year from brain-related diseases. In addition to the high cost of treatment, neurological diseases strongly affect the presence of these patients in social activities and their quality of life. The human brain, as the most complex organ, is affected by sex differences in all anatomical, functional, and biochemical aspects [35]. Sexual dimorphism plays critical roles in various parameters such as brain area volume, cell number and cytoarchitecture, neural functions, synaptic connectivity, perception, cognition, and memory at all stages of development [36, 37]. In sexually dimorphic non-gonadal tissues such as the brain, it has been shown that the development of neurons in the brain is influenced by a regulated combination of the secretion of sex hormones such as testosterone in men and estrogen in women and the function of X and Y chromosomes, which exert sex-specific effects on the development and differentiation of XX and XY neurons [38]. Furthermore, it has been found that the brain cells of men and women, independent of the secretion of sex hormones, follow distinct transcriptional patterns, which can be the cause of differences in the brain developmental pathways, brain function, and behaviors of males and females [39]. The BrainSpan atlas (www.brainspan.org) showed the transcriptional expression of several Y chromosome genes during various stages of male brain development (e.g. *SRY*, *RPS4Y1*, *ZFY*, *PCDH11Y*, *TBL1Y*, *PRKY*, *USP9Y*, *DDX3Y*, *UTY/KDM6C*, *TMSB4Y*, *NLGN4Y*, *HSFY*, *TXLNGY*, *KDM5D* and *EIF1AY*). Furthermore, Vakilian et al. demonstrated that the expression of several MSY genes including *RBMY1*, *EIF1AY*, *DDX3Y*, *HSFY1*, *BPY2*, *PCDH11Y*, *UTY*, *RPS4Y1*, *USP9Y*, *SRY*, *PRY*, and *ZFY* was significantly overexpressed during neural cell differentiation of NTERA-2, a human embryonal carcinoma cell line [40]. They also showed that DDX3Y knockdown inhibited neural cell differentiation of NTERA-2 through cell growth arrest at the G1/S phase and overexpression of pro-apoptotic proteins [40]. There is ample evidence that the prevalence, susceptibility, and progression to deficits in the dopamine system such as Parkinson’s disease (PD), attention-deficit hyperactivity disorder (ADHD), schizophrenia, and autism spectrum disorders (ASD), are higher in males than females [37].

Simunovic et al. performed a gene expression analysis on laser microdissected dopamine (DA) neurons from postmortem brains of sporadic PD male and female patients and showed that the major cellular pathways involved in PD pathogenesis such as oxidative phosphorylation, apoptosis, and synaptic transmission were more down-regulated in males compared to females. Results provided strong evidence on sex-specific dysregulation of gene expression in the pathogenesis of sporadic PD [41].

Dewing et al. showed the Y chromosome-linked, male-determining gene *SRY*, which is dominantly expressed in dopamine-abundant regions of the adult brain, directly regulates male-specific brain function. It modulates dopamine biosynthesis and subsequently affects voluntary movement in the male rodents, so it may increase the risk of disorders such as ASD and PD in males [42]. Lee et al. showed that nigral Sry expression persistently increased in animal and cell culture PD models and led to a male-specific mechanism of DA cell death. Reduction of nigral Sry expression by antisense oligonucleotides induced male-specific protective effects through the inhibition of DNA damage, mitochondrial degradation, and neuroinflammation in PD models [43]. These findings indicated that aside from the protective effects of female sex hormones, *Sry* up-regulation may also explain male bias in PD. In addition, it has been observed that mutations in Y chromosome genes such as NLGN4Y may be involved in the development of inherited diseases such as ASD [8]. NLGN4Y is a male-specific cell adhesion molecule belonging to the neuroligin (NLGN) family that plays a critical role in the formation of functional synapses and regulation of synaptic activity [44]. Thus, mutation or any failure in its translation or function of NLGN4Y can lead to the development of ASD. The *NLGN4Y* mutation in XY men, as well as the increased NLGN4Y expression in males with XYY have been reported to be directly associated with autism [45]. Bioinformatic analyses on ChIP-seq/chip and gene expression datasets have shown that SRY/SOX3 target genes regulate sex-specific developmental processes such as neurodevelopment and potentially could contribute to sex-biased neurodevelopmental disorders. Furthermore, exclusive SRY or SOX3 target genes were found to be more associated with the late gestational and postnatal periods. Analysis of co-expressed networks of SOX3/SRY target genes provided new evidence for the regulatory role of SOX3 in both sexes while SRY exclusively contributes to ASD male predisposition [46]. Loss of chromosome Y (LOY), a mosaic aneuploidy which mainly detected in circulating white blood cells, has been considered as one of the underlying causes of aging-related diseases [47, 48]. Dumanski et al. applied SNP-array and whole-genome next-generation sequencing (WGS) to detect and validate the level of LOY mosaicism in three independent studies of different types including a case–control study and two prospective studies. Results indicated that men with LOY in blood cells are more susceptible to Alzheimer’s disease (AD) [47]. Defective immunosurveillance as a result of extreme down-regulation of chromosome Y (EDY) could be a possible explanation for the association between LOY in blood cells and disease processes in other tissues [48].

The association of the Y chromosome and other neurological disorders such as ADHD and schizophrenia have also been investigated in a few studies [49, 50]. Although there are pieces of evidence that indicate the role of PCDH11Y in the susceptibility to psychiatric disorders, they were not supported by the study of Durand et al. in which the frequency of two *PCDH11Y* variants (F885V and K980) in males with different psychiatric disorders such as schizophrenia and ADHD was studied and no significant differences were observed in comparison with control populations [51].

## Y chromosome in cardiovascular diseases

Cardiovascular diseases (CVDs) are a group of conditions affecting the heart and blood vessels and are the leading cause of death globally [52]. Studies have been shown gender-specific phenotypes in cardiac physiology and pathophysiology [52, 53]. Incidence, frequency, and severity of coronary artery disease (CAD), as the most common type of CVD, is higher in men than in women [52]. The reason for sexual dimorphism in the prevalence of CVD is not fully understood, however, there are strong shreds of evidence that sex-specific hormones might impact the cardiac homeostasis in females and males resulting in estradiol-related protection and testosterone-associated vulnerability, respectively [53, 54]. In addition to the effective mechanisms of hormones and their receptors, these sexual dimorphisms might also be induced by sex chromosomes [55]. Studies have shown that the differences between the sequence, expression, and regulatory roles of sex chromosomal genes, independent of the gonad and its hormonal influence, result in cell autonomous sexual dimorphism [56, 57]. Comparison of the heart function between two mouse strains, C57BL/6J and C57BL/6J.Y^A/J^ (a chromosome-substituted C57BL/6J line in which the original MSY had been substituted for that from A/J mice) revealed that androgens alone are not sufficient to exert male-specific phenotypes in certain cardiac functions such as circadian rhythms and myocardial functional reserve. Genetic material from MSY was considered as a mandatory element to complete the functions of androgens [58].

A positive correlation has been reported between men diagnosed with Y polysomy and the risk of circulatory system death [59]. Population-based studies showed the risk of CAD increases in carriers of haplogroup I1 compared to other Y chromosome haplogroups, showing pleiotropic effects of the Y chromosome on susceptibility to CAD [5, 10]. The risk of atherosclerotic plaque and femoral artery bifurcations also increases in haplogroup k compared to other ones [60]. Genotyping of 11 MSY markers in three cohorts including 3233 British men showed the association between haplogroup I and increased risk of CAD [61]. Transcriptome-wide analysis of macrophages derived from 134 patients with premature myocardial infarction and 121 normal controls led to identify 30 differentially expressed pathways between haplogroup I1 and carriers of other haplogroups which were majorly involved in immunity, confirming the important role of inflammation in the pathogenesis of CAD [61]. Bloomer et al. showed that the expression of *UTY* and *PRKY* decreased in macrophages derived from men with haplogroup I lineage [62]. Down-regulation of *UTY* in macrophages led to changes in the expression of 59 CAD-related pathways [10]. These observations along with the animal model study indicate that inflammation can be considered as a missing link between the Y chromosome and CAD [5, 63].

The association of MSY genes with the risk factors of CVD including hypertension, circulating total cholesterol, LDL, and paternal history of cardiac diseases has been shown using gene single nucleotide polymorphisms [64–66]. Transcriptome analyses of heart tissues from healthy individuals and patients with non-ischemic cardiomyopathy and new-onset heart failure showed the differences in expression levels of sex chromosome genes. Y-chromosome-related transcripts including USP9Y, DDX3Y, RPS4Y1, and EIF1AY were overexpressed in males while the expression of two X-linked genes *XIST* and *ZFX* increased in females with new-onset heart failure [67]. The onset of sex-biased protein expression and sex disparities in heart tissue was observed in the early stages of embryonic development, before the gonad formation [68]. By taking advantage of human PSC, Meyfour et al. showed that Y chromosome genes are differentially expressed during cardiac development [6]. Among them, TBL1Y was overexpressed at the cardiac mesoderm stage, an opposite expression pattern to what was observed for its X counterpart, TBL1X [6]. The association of functional null mutations of *TBL1Y* with non-syndromic coarctation of the aorta confirmed its important role in the pathophysiology of CVD [69]. The necessity of KDM5D expression has been reported during the differentiation of human embryonic stem cells into cardiomyocytes. Down-regulation of KDM5D interrupted the cardiac differentiation by inhibiting cell cycle progression [70].

UTY and UTX/KDM6A belong to a subfamily of JmjC domain-containing proteins that catalyze the demethylation of *N*^ϵ^-methylated histone 3 lysine 27 (H3K27), an important mark for transcriptional repression [71]. Wang et al. reported that *UTX* knockout (KO) male embryonic stem cells (ESC) showed severe defects in mesoderm differentiation and induction of Brachyury [72]. Regarding the derivation of cardiomyocytes from mesoderm and the regulatory role of Brachyury in ESC differentiation into the mesoderm, the role of UTX in cardiac development can be concluded [72]. They also indicated that *UTY* can partially compensate *UTX* deficiency because male *UTX* KO mouse embryos expressed normal levels of UTY and survived until birth, while female *UTX* KO mice showed defects in embryonic cardiac development and Brachyury expression which led to embryonic lethality [72]. Lee et al. also confirmed the expression of *UTY* and *UTX* in developing mouse embryos [73]. Cardiac differentiation of *UTX*-null ESC revealed that UTX was necessary to activate the cardiac-specific gene program and expression of *UTY* was dependent on *UTX*. However, unlike male ESC, the expression of UTY was detected in *UTX*-null *UTX*^Δ/y^ male embryos, indicating the independent expression of *UTY* from *UTX* in male mouse embryos. This study also supported the compensatory role of UTY for its X homolog [73].

Several studies showed that mutations in the BCL6 corepressor (*BCOR*) gene located on the X chromosome could be responsible for different diseases such as Lenz microphthalmia syndrome and oculofaciocardiodental (OFCD) in which cardiac defect is one of the main predominate phenotypes [74, 75]. Zhu et al. reported a 7-month-old boy with Lenz microphthalmia/OFCD syndrome that had multiple defects such as glaucoma, cerebral white matter hypoplasia, and congenital heart defect. Genetic analysis showed a novel missense mutation (c.G1619A; p.R540Q) in *BCOR* [76]. Overexpression of *BCORP1* has been reported during cardiac differentiation of ESC into cardiomyocytes [6] thus like its X counterpart, *BCOR* may contribute to congenital anomalies.

## Y chromosome in autoimmune and infectious diseases

Y chromosome has been introduced as a regulatory element of immune cell transcriptome that is involved in susceptibility to autoimmune and infectious diseases [55, 77]. LOY analysis using SNP-arrays in sorted- and single-cells leukocytes showed that LOY in CD4^+^ T cells, granulocytes, and NK cells can be associated with different diseases such as AD and cancer. Furthermore, RNA-sequencing (RNA-Seq) of leukocytes demonstrated the LOY-associated transcriptional effect (LATE) on autosomal genes. LATE genes were majorly involved in immune functions, explaining how LOY in immune cells increases the risk for diseases [78].

Using chromosome Y-substituted mouse strains, it was shown that variation in copy number of *Sly* and *Rbmy* on the Y chromosome plays a potential role in susceptibility to and severity of autoimmune diseases including experimental allergic encephalomyelitis and myocarditis [77]. Gene expression analysis of circulating naïve CD4^+^ T cells from 37 patients with the clinically isolated syndrome (CIS), an early form of multiple sclerosis [79] and overlapping with data set obtained from CD4^+^ T cells of chromosome Y-substituted mouse strains led to identifying 440 genes common between mouse and human which were involved in central dogma, providing further evidence of a profound effect Y chromosome on susceptibility to autoimmune disease [77].

The role of *UTY* and its X homolog *UTX* has been determined in the production of proinflammatory cytokines. Kruidenier et al. introduced a small molecule catalytic site inhibitor that could selectively target the function of H3K27-specific JMJ subfamily and reduce the expression of inflammatory cytokines in LPS-induced macrophages [80]. Dysregulation of *UTY* is a characteristic of Y chromosome haplogroup l [62]. *UTY* encodes a histocompatibility antigen that plays a crucial role in the rejection of male stem cell transplantation [81].

Sex differences in susceptibility to infectious diseases have also been considered as a major challenge in dealing with these types of diseases ignored [82]. Although the influence of the sex hormones on the immune system is undeniable, the influence of sex chromosomes on susceptibility to infectious disease cannot be ignored [83]. Krementsov et al. showed that genetic variation in chromosome Y affects the survival following murine influenza A virus (IAV) infection. They showed that specific Y chromosome variants increase susceptibility to IAV in males and reinforce activation of proinflammatory IL-17-producing γδ T cells in lung tissue [83]. The association between Y chromosome variants and survival following infection with Coxsackievirus B3 virus (CVB3) has also been reported [84].

The AIDS progression and related death as well as resistance to highly active antiretroviral therapy (HAART) are faster in HIV-infected men with Y haplogroup I than other Y haplogroups [85]. A plethora of epidemiological studies has indicated sex disparities in COVID-19 vulnerability. The prevalence of infection and death is higher in men compared to women [86]. Delanghe et al. reported a marked correlation between COVID-19 prevalence and mortality with R1b-S116 haplotype frequency in the European population [87]. The correlation of the ancestry marker R1b1b2 with both infection and mortality of SARS-CoV-2 needs to be more investigated [88]. These results could be related to the regulatory role of Y chromosome genes in viral infections, and immune and inflammatory responses [5].

## Y chromosome and cancer

The impact of sex differences on the risks, incidence, and progression of various cancers has been reported in numerous studies [89, 90]. Cook and colleagues showed the male preference of cancer mortality in different cancer types [91]. Aberrant expression of Y chromosome genes may explain some mechanisms responsible for such sex differences in susceptibility and incidence of cancers [92]. Tricarico and colleagues emphasized a role for the differential activity of X- and Y-linked tumor-suppressor genes in males and females. Enzymatic and non-enzymatic activities of these epigenetic modulators profoundly change the expression of target genes [89].

## Liver cancer

Liver cancer is the fourth leading cause of global cancer death and the cause of over 700,000 death annually. Primary liver cancer is classified into different types; hepatocellular carcinoma (HCC, almost 85% of the cases), intrahepatic cholangiocarcinoma (ICC, 10–15% of cases), and also some other rare cases [93]. Etiologically HCC is correlated with a variety of factors like aflatoxin, smoking, heavy alcohol consumption, and especially Hepatitis B virus (HBV) infection [94]. It has been shown that sexual dimorphism is a risk factor for this disease and male cirrhotic patients are more susceptible to develop HCC than female patients [95]. HCC incidence in men is about 3–6 times more than in women [96], therefore, sex is a key factor for prognosis, aggressiveness, and treatment of this type of liver cancer. Although sex hormones like androgens and estrogens have been studied as a potential factor involved in hepatocyte development and enhancers of HCC proliferation, the exact molecular mechanism of this cancer is not fully understood. There is no reasonable evidence on the hormone response of HCC cells, and androgen and estrogen therapy did not indicate a beneficial effect on patients’ survival [97]. Furthermore, there is some evidence about the effect of the androgen receptor (AR) on the progression of hepatocarcinogenesis in patients carrying HBV and HCV [98]. The liver is an organ with sexual dimorphism in immune response, mitochondrial function, membrane lipid composition, and gene expression (99). Park et al. investigated the detailed genomic alterations in 5 Korean HCC cell lines using comparative genomic hybridization (CGH). Results showed significant loss of DNA copy number of cancer-related genes on the Y chromosome such as *TSPY*, *XKRY*, *PRY* in comparison with normal samples [100].

Aberrant expression of *TSPY*, *RBMY*, *SRY*, *VCY,* and the other Y chromosome genes has been reported to be involved in hepatocellular carcinogenesis [92, 101–104]. Kido and colleagues showed that *TGF2LY* and *VCY* were up-regulated in about 30% of HCC patients, while *DDX3Y*, *ZFY*, and *DAZ1* were down-regulated in about 70% of patients [92].

Dual functional roles of the Y-linked RBMY have been reported in hepatocarcinogenesis in different studies [101, 103, 105–107]. Tsuei et al. showed the expression of one to four different transcripts of RBMY including wild type and variants with N-terminal RRM deletion, C-terminal SRGY (serine–arginine–glycine–tyrosine) boxes deletion, or deletion of both domains in males with HCC and hepatoblastoma. Given that RBMY is a male germ cell-specific RNA-binding protein and it is not expressed in non-tumor liver counterparts, cirrhotic liver, and the other cancers, *RBMY* could be introduced as a new male-specific oncogene for liver cancer [103].

Western blot and immunohistochemistry (IHC) analyses of animal and human tissues showed that the Ser/Thr phosphorylated RBMY was only expressed in the cytoplasm of human and rodent fetal hepatocytes while its expression was inactivated in mature cells. However, cytoplasmic expression of RBMY was also observed in hepatic cancer stem cells and significantly associated with a poor prognosis and decreased survival rate in HCC patients. Mechanistically, cytoplasmic expression of RBMY leads to inactivation of glycogen synthase kinase 3β, translocation of β-catenin to the nucleus, and abnormal activation of the Wnt/β-catenin signaling pathway, thus facilitating the proliferation and cell cycle progression in HCC cells [106]. Down-regulation of *RBMY* reduced the transformation and anti-apoptotic ability of HepG2 cells, while expression of *RBMY* induced hepatocarcinogenesis in transgenic mice. In fact, RBMY increased AR activity and induced carcinogenic effects in hepatoma cell lines and human HCC tissues through down-regulation of AR inhibitory variant AR45. Therefore, regulation of AR activity can be considered as another mechanism of RBMY involvement in hepatocarcinogenesis [105].

Data mining of IHC analyses of HCC specimens also confirmed the oncogenic properties of *RBMY* in HCC, however, overexpression of *RBMY* in an HCC cell line HuH-7 and a hepatoblastoma cell line HepG2 showed an inhibitory effect on cell proliferation. Overexpression of *RBMY* in HuH-7 cell line led to down-regulation of the RAS/RAF/MAP and PIP3/AKT signaling pathways and abolished HCC development in a mouse liver cancer model. Altogether, Kido et al. concluded that the expression levels and spatiotemporal patterns of RBMY define the tumor-suppressing or oncogenic roles of RBMY during oncogenic processes. It seems that RBMY functions as a male-specific tumor suppressor at early stages of HCC development and can suppress cell proliferation and pro-oncogenic pathways. However, after surviving and adapting tumor cells in proliferative mode, RBMY acts as a proto-oncogene and induces its chronic effects to promote HCC progression [107].

The association of *TSPY* as a proto-oncogene and inhibitor of anti-oncogenic genes has been reported with a poor prognosis of HCC in men [102]. *TSPY* gene is located within the gonadoblastoma locus on the Y chromosome (GBY) with over 30 tandemly repeats, which increases the risk of gonadoblastoma development in XY patients with disorders of sexual development [108–111]. Expression of this gene is frequently observed in some somatic cancers such as liver cancer [92, 112]. Its overexpression resulted in increased cell proliferation and tumor growth in HCC cases through the suppression of anti-oncogenic genes [111, 113]. It has been reported that cell-cycle regulators and cell division factors like BUB1, CDC25B, CDC45, CENPA, PRC1, PRIM1, RRM2, SPC24, and growth factor receptors like ADGRD1 and HMMR are up-regulated by the *TSPY* gene [102]. Shirakawa and colleagues showed the co-expression of *TSPY* and Glypican-3 (*GPC3*) as a sensitive and specific biomarker of HCC [114]. Kido and colleagues identified a TSPY co-expression network (TCN) which activated in 30% of males with HCC [101]. Ectopic activation of *TSPY* and/or inactivation of its X homolog (*TSPX*) as a tumor suppressor could explain sexual dimorphisms in HCC [112].

There is also some evidence on the oncogenicity of the SRY and the formation of cancer stem cells in male HCC [115]. In an in vivo study, down-regulation of *Sry* resulted in lower malignancy, invasiveness, and tumorigenesis of rHCC cells via the inhibition of Sgf29. Sgf29 is a subunit of the SAGA (Spt–Ada–Gcn5 acetyltransferase) complex that is required to bind tri-methylated lysine-4 of histone H3 (H3K4me3). Studies have been revealed that SRY is the upstream regulator of this gene and up-regulation of *Sgf29* induces tumorigenicity and metastasis through the c-Myc-mediated malignant transformation [116, 117]. Furthermore, data mining of RNA-Seq data of 27 male tumor/non-tumor paired samples from The Cancer Genome Atlas (TCGA) showed that *DAZ1* and *BPY2* are frequently down-regulated in HCC patients [92].

## Prostate cancer

Prostate cancer (PC) is a genetically heterogeneous disease and genetic factors play crucial roles in the development of this cancer [118]. It is the second most common cancer and the fifth leading cause of malignancy in men worldwide [119]. It is also the cause of over 3% of death caused by cancer among men [120]. Changes in the Y chromosome genes are likely to be involved in the development and progression of PC. Genomic instability of Y chromosome specific repeated DNA family (DYZ1) has been observed in individuals with PC and it can be used as a marker [121]. Although Y losses occur at high rates in most cancer types, LOY is a rare event in PC [122]. Loss of *SRY*, *ZFY*, *BPY1*, *KDM5D*, *RBMY*, *BPY2,* and other MSY genes has been observed in high grades and advanced stages of this disease [123, 124]. Array-based CGH on prostate tumors and PC cell lines showed that loss of *TSPY* gene copies is associated with an increased risk of PC [125]. Furthermore, specific loci on the Y chromosome can be associated with PC. Some loci increase the incidence of PC and some others decrease it [126–129]. In a study conducted by Nargessi et al., four Y-linked short tandem repeats (STRs), including DYS388, DYS435, DYS437, and DYS439 were genotyped in Malaysian males with PC and healthy controls using a Genetic Analysis System. Results revealed that allele 12 of DYS388, allele 14 of DYS439, or haplotype CAAA are associated with susceptibility to develop PC, and Y-lineages with allele 10 of DYS388 or haplotype AABC are more resistant to the disease. Therefore, these DYS loci as well as haplotypes could be used as a screening method to predict PC susceptibility among Malaysian males [126]. Furthermore, six aberrant DNA methylation sites on the Y chromosome were found in PC tissues, of which cg05163709 site methylation was significantly correlated with PC and was presented as a potential diagnostic biomarker with high specificity and sensitivity [129].

Reverse transcription-PCR (RT-PCR) analysis of Y chromosome genes in a panel of samples diagnosed with low/high grade prostate adenocarcinoma and benign prostatic hyperplasia (BPH), as well as PC cell lines showed the differential expression patterns of Y chromosome genes such as *SRY*, *PRY*, *TSPY*, *RBMYIH*, *SMCY*, *ZFY* and *EIF1AY* in PC [130, 131]. These results indicated that Y chromosome genes might be either involved in or influenced by oncogenic processes governing PC development and progression. Co-expression networks were reconstructed using an available microarray dataset on normal and different stages of PC tissues, which was deposited with the NCBI Gene Expression Omnibus (GEO), to investigate the role of Y chromosome genes in PC biology. Network analysis led to identify 18 PC-related pathways in which 22 Y chromosome genes were enriched. *CYORF15B*, *DAZ4,* and *PRY2* were up-regulated while *RBMY1J*, *USP9Y*, *DDX3Y,* and *KDM5D* showed an opposite expression pattern and decreased during PC progression [132].

*TSPY*, a Y chromosome-linked oncogene, is frequently activated in PC and its expression is correlated with the poor prognosis of PC [133, 134]. It can shorten the G2/M stage and accelerate cell proliferation [135]. Other studies showed that AR binds to the *TSPY* promoter and enhances its transcription through the regulation of DNA methylation in PC cells [133]. TSPY and its X-located homolog (TSPX) competitively bind to the AR and play opposing roles in the transactivation functions of AR and AR-Variants which can explain the pathogenesis of male-specific PC as well as sexual dimorphisms in the health and diseases of men [111].

KDM5D, a male-specific histone demethylase has been introduced as a tumor suppressor gene in different studies [136–139]. The knockdown of two different isoforms of KDM5D using the short interfering RNA (siRNA) approach confirmed its tumor suppressor role in a PC cell line [136].

Fluorescence in situ hybridization (FISH) analysis showed that the deletions on Y chromosome could explain the cause of decreased KDM5D expression in PC cells. Furthermore, *KDM5D* knockdown led to aggressive PC by altering the expression of target genes such as cell cycle regulators. ChIP-sequencing and motif analyses of KDM5D-binding sites confirmed that KDM5D as a chromatin modifier binds to promoter regions with co-enrichment of the motifs of critical transcription factors such as the E2F family and MYBL2 that regulate the cell cycle [139]. Furthermore, KDM5D levels were highly reduced in metastatic prostate tumors compared with normal tissues and primary prostate tumors. KDM5D suppresses invasion-associated genes including *MMP1*, *MMP2*, *MMP3*, *MMP7*, and *Slug* in PC cells in vivo and in vitro through H3K4 demethylation [140]. Komura et al. showed the crucial role of AR signaling in the sensitivity of PC cell lines, LNCaP and LAPC4, to docetaxel. Docetaxel is prescribed as an important treatment option for patients with metastatic castration-resistant PC. RNA-Seq followed by functional analyses revealed that KDM5D can be considered as a potential mediator of docetaxel sensitivity in the presence of dihydrotestosterone (DHT). Mechanistically KDM5D binds to AR and controls its transcriptional activity by demethylating H3K4me3 active transcriptional marks [138]. They showed that serine/threonine protein kinase ATR inhibitors could be a new therapeutic approach in aggressive prostate tumors deficient in KDM5D [139].

LNCaP and LAPC4 long noncoding RNAs (lncRNAs) have been shown to get involved in critical physiological and pathological processes, such as PC [141]. In a recent study, it has been shown that lncRNA TTTY15 located in the AZFa region of the Y chromosome, is significantly up-regulated in PC tissues compared to normal ones [142]. Moreover, knockout of Y-chromosomal lncRNA TTTY15 using CRISPR/Cas9 technologies resulted in the inhibition of prostate cell growth and migration in vitro and in vivo, introducing lncRNA TTTY15 as a therapeutic target for PC [142].

## Germ cell tumors

Testicular germ cell tumor (TGCT) is the most common malignancy in men ages 15 to 35 [143].

Understanding the etiology and pathogenesis of TGCTs has received considerable attention due to their rising rate [144]. TGCTs have two distinct subtypes including seminomatous and non-seminomatous groups which are characterized by different molecular and histological patterns [145]. The involvement of genetic factors on the Y chromosome in the development of TGCTs has been investigated in various studies [146, 147]. Y microdeletion gr/gr has been introduced as a rare, low-penetrance allele that confers susceptibility to TGCT. Results from the largest European study showed that the *gr/gr* deletion increases the risk of TGCT independently from altered spermatogenesis [146]. *gr/gr* deletion is accompanied by loss of *DAZ*, *BPY2*, and *CDY1* genes, indicating the suppressor roles of these genes in TGCT development [148]. Quantitative PCR (qPCR) by 15 probes spanning the Y chromosome on blood- and buccal-derived DNA samples from two case–control studies including the Familial Testicular Cancer Study (FTC) and the Servicemen’s Testicular Tumor Environmental and Endocrine Determinants Study (STEED) revealed the possible association between mLOY and familial TGCT. However, there was not a significant difference in target to reference (T/R) ratio between TGCT cases and controls for the STEED samples. *ZFY*, *AMELY*, *USP9Y*, *DDX3Y*, *TMSB4Y*, *NLGN4Y*, *CYorf15A*, *CYorf15B*, and *EIF1AY* were markers that showed a significant T/R ratio in FTC samples [149].

Gonadoblastoma is the precursor of invasive TGCTs in dysgenetic gonads. GBY known as an oncogenic locus appears to be involved in TGCT development. IHC results showed that *TSPY* is ectopically and abundantly expressed not only in gonadoblastoma tissues, but also in TGCTs, including the premalignant precursor (carcinoma in situ), seminoma, and non-seminomas [150]. Several studies reported aberrant expression of *TSPY* as the putative gene of GBY, in gonadoblastomas and TGCTs [113, 150, 151]. Protein and gene expression analyses in TGCT tissues compared to normal samples indicated that TSPY co-expressed with proliferative markers such as Ki-67, cyclin B1, and germ cell tumor markers such as PLAP**,** OCT4, and c-KIT [150]. Overexpression of *TSPY* in Hela and NIH3T3 cells as well as in vivo analyses showed that TSPY could enhance cell proliferation and tumorigenesis. Mechanistically, the shortening of the G_2_/M transition in overexpressed *TSPY* cells was associated with an early degradation of the mitotic cyclin B1. Degradation of cyclin B1 is required to exit mitosis [113]. Promoter assay and functional domain analyses showed that TSPY is co-localized with AR in the promoters of the endogenous androgen-responsive genes and exacerbates AR functions. Considering the role of AR in cancer progression, the oncogenic role of *TSPY* can be concluded [111].

## Other cancers

Although mLOY has been reported as the most frequent somatic variant in different cancers in males, its phenotypic consequences are complex and ambiguous [152]. In a population-based study, Qin et al. proposed a “two-sides” model for the role of LOY in lung cancer in which genetically defined mLOY decreased the risk of lung cancer and predicted a better prognosis while aberrant LOY caused by environmental factors like smoking exerted an effect on lung carcinogenesis [153].

Transcriptome analysis of tumor and matched unaffected pulmonary tissues from patients with non-small-cell lung cancer (NSCLC) led to identify sex-specific co-expression networks. Results showed that partial losses of the Y chromosome, particularly *KDM5D* deficiency at the heart of the co-expression network increases the risk of death in NSCLC male patients, thus may contribute to sexual dimorphism in lung cancer [154]. Analysis of sequencing read coverage of 20 MSY genes and RNA-seq data obtained from normal and tumor tissues also showed that the expression of epigenetic modifiers KDM5D and/or KDM6C is reduced due to LOY in clear cell renal cell carcinoma (ccRCC) [155]. The mechanism of action of *KDM5D* as a tumor suppressor gene has been investigated in gastric cancer (GC) [156]. IHC staining of GC and normal tissues showed the decreased expression of KDM5D in GC. Down-regulation of *KDM5D* accelerated the migration and invasion of GC cells by activating epithelial–mesenchymal transition (EMT). Mechanistically, decreased expression of *KDM5D* induces the expression of cullin 4A (*CUL4A*), which in turn leads to the overexpression of *ZEB1* (EMT inducer) and down-regulation of *p21* and *p53* [156]. It seems that upstream oncogenic factors such as ETS variant 4 (ETV4) and mirR-4661-5p decrease the expression of *KDM5D*, which subsequently results in the activation of downstream oncogenes such as Methionyl‐tRNA synthetase 2 (*Mars2*) in GC [157, 158].

DNA analyses of the peripheral blood sample from male patients with PC and colorectal cancer (CC), and healthy controls showed that LOY is a more significant predictor of cancer presence than age [159, 160]. In addition, Agahozo et al. performed ICH and FISH analyses using an X and Y probe to evaluate the prevalence of LOY in male breast cancer (BC). They introduced LOY as an early indicator of male breast carcinogenesis, particularly in estrogen-receptor (ER) and progesterone receptor (PR) negative tumors [161]. Westra et al. assessed the risk of Barrett’s esophagus (BE) and esophageal adenocarcinoma (EAC) development among six Y-chromosomal haplogroups including DE, F (xJ, xK), K (xP), J, P (xR1a), and R1a in a white male population. F haplogroup was found to predispose BE patients to cancer development while R1a and K haplogroups were determined as protective factors against BE development [162]. The correlation of LOY with poor prognosis of EAC was also demonstrated by using Y chromosome specific fluorescence in-situ probes [163]. In addition to the above-mentioned studies, a significantly higher frequency of LOY has been reported in blood cells of patients with colorectal, head and neck, bladder, leukemia, and pancreatic cancers compared to healthy individuals [160, 164–167].

The role of Y chromosome-linked noncoding RNAs in cancer progression and suppression is also remarkable. Low *TTTY15* expression resulted in down-regulation of TBX4 and a worse prognosis of NSCLC patients [168]. Brownmiller and colleagues also showed the role of Y chromosome lncRNAs in the radiation response of male NSCLC cells [169]. A Y-linked lncRNA, LINC00278 that encodes a Yin Yang 1 (YY1)-binding micropeptide (YY1BM), is down-regulated in male esophageal squamous cell carcinoma (ESCC). YY1BM, which functions as a tumor suppressor, binds to multifunctional transcription factor YY1 and blocks its interaction to AR, thus decreasing the expression of Eukaryotic Elongation Factor 2 Kinase (eEF2K) and inducing apoptosis in ESCC. Cigarette smoking negatively affects m6A modification of LINC00278 and YY1BM translation and leads to male ESCC progression [170].

## Conclusion

Although most sex differences in occurrence and prevalence of diseases have been associated with the function of sex hormones, molecular studies have assigned a hormone-independent role to the differential expression of genes, especially those located on sex chromosomes. Y chromosome genes independently and/or in conjunction with sex hormones, beyond their X-linked collective tasks determine the male-specific characteristics. In this review, we highlighted major recent findings on the contribution of Y chromosome genes to disease susceptibility to various human diseases and showed that how LOY and translation/function failure of Y chromosome genes can affect the pathogenesis of male-specific diseases.

Despite the vast investigation, little knowledge exists on the molecular mechanisms involved in these sex disparities. This might have been originated from the biological limitations and/or experimental issues such as low expression of MSY genes in rare organs or cell types, high similarity with their X counterparts, hormone effects on intracellular processes, and the absence of mixed-sex experimental groups in cellular, animal, and human studies. In the human Y chromosome proteome project, as a part of the Chromosome-Centric Human Proteome Project (C-HPP), the function of MSY proteins was explored in organ development by taking advantage of PSCs, which are capable of differentiation into all cell types of the human body [171]. We believe that hormone-free systems like PSC and their derivatives as well as organoids, which are in vitro generated copies of human organs, can facilitate the mechanistic studies to explore the role of Y chromosome genes in health and disease and provide novel insights into gender disparity and sex-specific therapeutic strategies for diseases.

## Data Availability

Not applicable.

## References

[1] Skaletsky H, Kuroda-Kawaguchi T, Minx PJ, Cordum HS, Hillier L, Brown LG (2003). The male-specific region of the human Y chromosome is a mosaic of discrete sequence classes. Nature.

[2] Bellott DW, Hughes JF, Skaletsky H, Brown LG, Pyntikova T, Cho T-J (2014). Mammalian Y chromosomes retain widely expressed dosage-sensitive regulators. Nature.

[3] Colaco S, Modi D (2018). Genetics of the human Y chromosome and its association with male infertility. Reprod Biol Endocrinol.

[4] Meyfour A, Pooyan P, Pahlavan S, Rezaei-Tavirani M, Gourabi H, Baharvand H (2017). Chromosome-centric human proteome project allies with developmental biology: a case study of the role of Y chromosome genes in organ development. J Proteome Res.

[5] Maan AA, Eales J, Akbarov A, Rowland J, Xu X, Jobling MA (2017). The Y chromosome: a blueprint for men's health?. Eur J Hum Genet.

[6] Meyfour A, Ansari H, Pahlavan S, Mirshahvaladi S, Rezaei-Tavirani M, Gourabi H (2017). Y chromosome missing protein, TBL1Y, may play an important role in cardiac differentiation. J Proteome Res.

[7] Skuse DH (2000). Imprinting, the X-chromosome, and the male brain: explaining sex differences in the liability to autism. Pediatr Res.

[8] Serajee FJ, Mahbubul HA (2009). Association of Y chromosome haplotypes with autism. J Child Neurol.

[9] Goulmy E, Termijtelen A, Bradley B, Van Rood J (1977). Y-antigen killing by T cells of women is restricted by HLA. Nature.

[10] Eales JM, Maan AA, Xu X, Michoel T, Hallast P, Batini C (2019). Human Y chromosome exerts pleiotropic effects on susceptibility to atherosclerosis. Arterioscler Thromb Vasc Biol.

[11] Arnold AP (2012). The end of gonad-centric sex determination in mammals. Trends Genet.

[12] Al-Achkar W, Wafa A, Moassass F (2013). Cytogenetic abnormalities and Y-chromosome microdeletions in infertile Syrian males. Biomed Rep.

[13] Mierla D, Malageanu M, Tulin R, Albu D (2015). Prevalence of chromosomal abnormalities in infertile couples in Romania. Balkan J Med Genet.

[14] Kalantari H, Asia S, Totonchi M, Vazirinasab H, Mansouri Z, Moradi SZ (2014). Delineating the association between isodicentric chromosome Y and infertility: a retrospective study. Fertil Steril.

[15] Bansal SK, Gupta G, Rajender S (2016). Y chromosome b2/b3 deletions and male infertility: a comprehensive meta-analysis, trial sequential analysis and systematic review. Mutat Res Rev Mutat Res.

[16] Vogt PH (2005). Azoospermia factor (AZF) in Yq11: towards a molecular understanding of its function for human male fertility and spermatogenesis. Reprod Biomed Online.

[17] Vogt PH, Bender U, Deibel B, Kiesewetter F, Zimmer J, Strowitzki T (2021). Human AZFb deletions cause distinct testicular pathologies depending on their extensions in Yq11 and the Y haplogroup: new cases and review of literature. Cell Biosci.

[18] Krausz C, Degl'Innocenti S, Nuti F, Morelli A, Felici F, Sansone M (2006). Natural transmission of USP9Y gene mutations: a new perspective on the role of AZFa genes in male fertility. Hum Mol Genet.

[19] Stouffs K, Lissens W, Verheyen G, Van Landuyt L, Goossens A, Tournaye H (2004). Expression pattern of the Y-linked PRY gene suggests a function in apoptosis but not in spermatogenesis. Mol Hum Reprod.

[20] Sato Y, Yoshida K, Shinka T, Nozawa S, Nakahori Y, Iwamoto T (2006). Altered expression pattern of heat shock transcription factor, Y chromosome (HSFY) may be related to altered differentiation of spermatogenic cells in testes with deteriorated spermatogenesis. Fertil Steril.

[21] Vinci G, Raicu F, Popa L, Popa O, Cocos R, McElreavey K (2005). A deletion of a novel heat shock gene on the Y chromosome associated with azoospermia. Mol Hum Reprod.

[22] Reijo R, Lee T-Y, Salo P, Alagappan R, Brown LG, Rosenberg M (1995). Diverse spermatogenic defects in humans caused by Y chromosome deletions encompassing a novel RNA-binding protein gene. Nat Genet.

[23] Reynolds N, Cooke HJ (2005). Role of the DAZ genes in male fertility. Reprod Biomed Online.

[24] Kee K, Angeles VT, Flores M, Nguyen HN, Reijo Pera RA (2009). Human DAZL, DAZ and BOULE genes modulate primordial germ-cell and haploid gamete formation. Nature.

[25] Lahn BT, Tang ZL, Zhou J, Barndt RJ, Parvinen M, Allis CD (2002). Previously uncharacterized histone acetyltransferases implicated in mammalian spermatogenesis. Proc Natl Acad Sci.

[26] Tse J, Wong E, Cheung A, O W, Tam P, Yeung W (2003). Specific expression of VCY2 in human male germ cells and its involvement in the pathogenesis of male infertility. Biol Reprod.

[27] Wong EY, Jenny Y, Yao K-M, Tam P-C, Yeung WS (2002). VCY2 protein interacts with the HECT domain of ubiquitin-protein ligase E3A. Biochem Biophys Res Commun.

[28] Ahmadi Rastegar D, Sharifi Tabar M, Alikhani M, Parsamatin P, Sahraneshin Samani F, Sabbaghian M (2015). Isoform-level gene expression profiles of human Y chromosome azoospermia factor genes and their X chromosome paralogs in the testicular tissue of non-obstructive azoospermia patients. J Proteome Res.

[29] Chen S, Zhang Q, Chu L, Chang C, Chen Y, Bao Z (2021). Comprehensive copy number analysis of Y chromosome-linked loci for detection of structural variations and diagnosis of male infertility. J Hum Genet.

[30] Vodicka R, Vrtel R, Dusek L, Singh AR, Krizova K, Svacinova V (2007). TSPY gene copy number as a potential new risk factor for male infertility. Reprod Biomed Online.

[31] Zhu Y, Hu L, Cao D, Ou X, Jiang M (2020). Chromosomal microarray analysis of infertile men with azoospermia factor microdeletions. Gene.

[32] Yan Y, Yang X, Liu Y, Shen Y, Tu W, Dong Q (2017). Copy number variation of functional RBMY1 is associated with sperm motility: an azoospermia factor-linked candidate for asthenozoospermia. Hum Reprod.

[33] Noordam MJ, Westerveld GH, Hovingh SE, van Daalen SK, Korver CM, van der Veen F (2011). Gene copy number reduction in the azoospermia factor c (AZFc) region and its effect on total motile sperm count. Hum Mol Genet.

[34] Suzuki E, Kobori Y, Katsumi M, Ushijima K, Uchiyama T, Okada H (2020). Copy-number analysis of Y-linked loci in young men with non-obstructive azoospermia: implications for the rarity of early onset mosaic loss of chromosome Y. Reprod Med Biol.

[35] Grgurevic N, Majdic G (2016). Sex differences in the brain—an interplay of sex steroid hormones and sex chromosomes. Clin Sci.

[36] Johansson MM, Lundin E, Qian X, Mirzazadeh M, Halvardson J, Darj E (2016). Spatial sexual dimorphism of X and Y homolog gene expression in the human central nervous system during early male development. Biol Sex Differ.

[37] Loke H, Harley V, Lee J (2015). Biological factors underlying sex differences in neurological disorders. Int J Biochem Cell Biol.

[38] Carruth LL, Reisert I, Arnold AP (2002). Sex chromosome genes directly affect brain sexual differentiation. Nat Neurosci.

[39] Sekido R (2014). The potential role of SRY in epigenetic gene regulation during brain sexual differentiation in mammals. Adv Genet.

[40] Vakilian H, Mirzaei M, Sharifi Tabar M, Pooyan P, Habibi Rezaee L, Parker L (2015). DDX3Y, a male-specific region of Y chromosome gene, may modulate neuronal differentiation. J Proteome Res.

[41] Simunovic F, Yi M, Wang Y, Stephens R, Sonntag KC (2010). Evidence for gender-specific transcriptional profiles of nigral dopamine neurons in Parkinson disease. PLoS ONE.

[42] Dewing P, Chiang CW, Sinchak K, Sim H, Fernagut P-O, Kelly S (2006). Direct regulation of adult brain function by the male-specific factor SRY. Curr Biol.

[43] Lee J, Pinares-Garcia P, Loke H, Ham S, Vilain E, Harley VR (2019). Sex-specific neuroprotection by inhibition of the Y-chromosome gene, SRY, in experimental Parkinson's disease. Proc Natl Acad Sci USA.

[44] Bottos A, Rissone A, Bussolino F, Arese M (2011). Neurexins and neuroligins: synapses look out of the nervous system. Cell Mol Life Sci.

[45] Zhang C, Milunsky JM, Newton S, Ko J, Zhao G, Maher TA (2009). A neuroligin-4 missense mutation associated with autism impairs neuroligin-4 folding and endoplasmic reticulum export. J Neurosci.

[46] Tahira AC, Barbosa AR, Feltrin ASA, Gastaldi VD, de Toledo VHC, de Carvalho Pereira JG (2019). Putative contributions of the sex chromosome proteins SOX3 and SRY to neurodevelopmental disorders. Am J Med Genet B Neuropsychiatr Genet.

[47] Dumanski JP, Lambert J-C, Rasi C, Giedraitis V, Davies H, Grenier-Boley B (2016). Mosaic loss of chromosome Y in blood is associated with Alzheimer disease. Am J Hum Genet.

[48] Caceres A, Jene A, Esko T, Perez-Jurado LA, Gonzalez JR (2020). Extreme downregulation of chromosome Y and Alzheimer's disease in men. Neurobiol Aging.

[49] Bache WK, DeLisi LE (2018). The sex chromosome hypothesis of schizophrenia: alive, dead, or forgotten? A commentary and review. Mol Neuropsychiatry.

[50] Crow TJ (2013). The XY gene hypothesis of psychosis: origins and current status. Am J Med Genet B Neuropsychiatr Genet.

[51] Durand CM, Kappeler C, Betancur C, Delorme R, Quach H, Goubran-Botros H (2006). Expression and genetic variability of PCDH11Y, a gene specific to Homo sapiens and candidate for susceptibility to psychiatric disorders. Am J Med Genet B Neuropsychiatr Genet.

[52] Molina E, Clarence EM, Ahmady F, Chew GS, Charchar FJ (2016). Coronary artery disease: why we should consider the Y chromosome. Heart Lung Circ.

[53] Regitz-Zagrosek V, Oertelt-Prigione S, Seeland U, Hetzer R (2010). Sex and gender differences in myocardial hypertrophy and heart failure. Circ J.

[54] Blenck CL, Harvey PA, Reckelhoff JF, Leinwand LA (2016). The importance of biological sex and estrogen in rodent models of cardiovascular health and disease. Circ Res.

[55] Regitz-Zagrosek V, Kararigas G (2017). Mechanistic pathways of sex differences in cardiovascular disease. Physiol Rev.

[56] Arnold AP (2017). Y chromosome’s roles in sex differences in disease. Proc Natl Acad Sci USA.

[57] Snell DM, Turner JMA (2018). Sex chromosome effects on male-female differences in mammals. Curr Biol.

[58] Praktiknjo SD, Picard S, Deschepper CF (2016). Comparisons of chromosome Y-substituted mouse strains reveal that the male-specific chromosome modulates the effects of androgens on cardiac functions. Biol Sex Differ.

[59] Higgins CD, Swerdlow AJ, Schoemaker MJ, Wright AF, Jacobs PA (2007). Mortality and cancer incidence in males with Y polysomy in Britain: a cohort study. Hum Genet.

[60] Voskarides K, Hadjipanagi D, Papazachariou L, Griffin M, Panayiotou AG (2014). Evidence for contribution of the y chromosome in atherosclerotic plaque occurrence in men. Genet Test Mol Biomarkers.

[61] Charchar FJ, Bloomer LD, Barnes TA, Cowley MJ, Nelson CP, Wang Y (2012). Inheritance of coronary artery disease in men: an analysis of the role of the Y chromosome. Lancet.

[62] Bloomer LD, Nelson CP, Eales J, Denniff M, Christofidou P, Debiec R (2013). Male-specific region of the Y chromosome and cardiovascular risk: phylogenetic analysis and gene expression studies. Arterioscler Thromb Vasc Biol.

[63] Khan SI, Andrews KL, Jennings GL, Sampson AK, Chin-Dusting JPF (2019). Y chromosome, hypertension and cardiovascular disease: is inflammation the answer?. Int J Mol Sci.

[64] Shankar RR, Charchar FJ, Eckert GJ, Saha C, Tu W, Dominiczak AF (2007). Studies of an association in boys of blood pressure and the Y chromosome. Am J Hypertens.

[65] Charchar FJ, Tomaszewski M, Lacka B, Zakrzewski J, Zukowska-Szczechowska E, Grzeszczak W (2004). Association of the human Y chromosome with cholesterol levels in the general population. Arterioscler Thromb Vasc Biol.

[66] Ellis JA, Stebbing M, Harrap SB (2000). Association of the human Y chromosome with high blood pressure in the general population. Hypertension.

[67] Heidecker B, Lamirault G, Kasper EK, Wittstein IS, Champion HC, Breton E (2010). The gene expression profile of patients with new-onset heart failure reveals important gender-specific differences. Eur Heart J.

[68] Shi W, Sheng X, Dorr KM, Hutton JE, Davies HA, Andrade TD, et al. Cardiac sex differences are established prior to gonad formation. bioRxiv. 2020.

[69] Tagariello A, Breuer C, Birkner Y, Schmidt S, Koch A, Cesnjevar R (2012). Functional null mutations in the gonosomal homologue gene TBL1Y are associated with non-syndromic coarctation of the aorta. Curr Mol Med.

[70] Meyfour A, Pahlavan S, Ansari H, Baharvand H, Salekdeh GH (2019). Down-regulation of a male-specific H3K4 demethylase, KDM5D, impairs cardiomyocyte differentiation. J Proteome Res.

[71] Walport LJ, Hopkinson RJ, Vollmar M, Madden SK, Gileadi C, Oppermann U (2014). Human UTY(KDM6C) is a male-specific N-methyl lysyl demethylase. J Biol Chem.

[72] Wang C, Lee JE, Cho YW, Xiao Y, Jin Q, Liu C (2012). UTX regulates mesoderm differentiation of embryonic stem cells independent of H3K27 demethylase activity. Proc Natl Acad Sci USA.

[73] Lee S, Lee JW, Lee SK (2012). UTX, a histone H3-lysine 27 demethylase, acts as a critical switch to activate the cardiac developmental program. Dev Cell.

[74] Hilton E, Johnston J, Whalen S, Okamoto N, Hatsukawa Y, Nishio J (2009). BCOR analysis in patients with OFCD and Lenz microphthalmia syndromes, mental retardation with ocular anomalies, and cardiac laterality defects. Eur J Hum Genet.

[75] Ng D, Thakker N, Corcoran CM, Donnai D, Perveen R, Schneider A (2004). Oculofaciocardiodental and Lenz microphthalmia syndromes result from distinct classes of mutations in BCOR. Nat Genet.

[76] Zhu X, Dai FR, Wang J, Zhang Y, Tan ZP, Zhang Y (2015). Novel BCOR mutation in a boy with Lenz microphthalmia/oculo-facio-cardio-dental (OFCD) syndrome. Gene.

[77] Case LK, Wall EH, Dragon JA, Saligrama N, Krementsov DN, Moussawi M (2013). The Y chromosome as a regulatory element shaping immune cell transcriptomes and susceptibility to autoimmune disease. Genome Res.

[78] Dumanski JP, Halvardson J, Davies H, Rychlicka-Buniowska E, Mattisson J, Moghadam BT (2021). Immune cells lacking Y chromosome show dysregulation of autosomal gene expression. Cell Mol Life Sci.

[79] Corvol JC, Pelletier D, Henry RG, Caillier SJ, Wang J, Pappas D (2008). Abrogation of T cell quiescence characterizes patients at high risk for multiple sclerosis after the initial neurological event. Proc Natl Acad Sci USA.

[80] Kruidenier L, Chung CW, Cheng Z, Liddle J, Che K, Joberty G (2012). A selective jumonji H3K27 demethylase inhibitor modulates the proinflammatory macrophage response. Nature.

[81] Vogt MH, Goulmy E, Kloosterboer FM, Blokland E, de Paus RA, Willemze R (2000). UTY gene codes for an HLA-B60-restricted human male-specific minor histocompatibility antigen involved in stem cell graft rejection: characterization of the critical polymorphic amino acid residues for T-cell recognition. Blood.

[82] Klein SL, Hodgson A, Robinson DP (2012). Mechanisms of sex disparities in influenza pathogenesis. J Leukoc Biol.

[83] Krementsov DN, Case LK, Dienz O, Raza A, Fang Q, Ather JL (2017). Genetic variation in chromosome Y regulates susceptibility to influenza A virus infection. Proc Natl Acad Sci USA.

[84] Case LK, Toussaint L, Moussawi M, Roberts B, Saligrama N, Brossay L (2012). Chromosome Y regulates survival following murine coxsackievirus b3 infection. G3 Genes Genomes Genet.

[85] Sezgin E, Lind JM, Shrestha S, Hendrickson S, Goedert JJ, Donfield S (2009). Association of Y chromosome haplogroup I with HIV progression, and HAART outcome. Hum Genet.

[86] Vahidy FS, Pan AP, Ahnstedt H, Munshi Y, Choi HA, Tiruneh Y (2021). Sex differences in susceptibility, severity, and outcomes of coronavirus disease 2019: cross-sectional analysis from a diverse US metropolitan area. PLoS ONE.

[87] Delanghe JR, De Buyzere ML, De Bruyne S, Van Criekinge W, Speeckaert MM (2020). The potential influence of human Y-chromosome haplogroup on COVID-19 prevalence and mortality. Ann Oncol.

[88] Ibrahim M, Salih A (2021). The Y chromosome ancestry marker R1b1b2: a surrogate of the SARS-CoV-2 population affinity. Hum Genome Var.

[89] Tricarico R, Nicolas E, Hall MJ, Golemis EA (2020). X- and Y-linked chromatin-modifying genes as regulators of sex-specific cancer incidence and prognosis. Clin Cancer Res.

[90] Fajkovic H, Halpern JA, Cha EK, Bahadori A, Chromecki TF, Karakiewicz PI (2011). Impact of gender on bladder cancer incidence, staging, and prognosis. World J Urol.

[91] Cook MB, McGlynn KA, Devesa SS, Freedman ND, Anderson WF (2011). Sex disparities in cancer mortality and survival. Cancer Epidemiol Biomarkers Prev.

[92] Kido T, Lau YF (2015). Roles of the Y chromosome genes in human cancers. Asian J Androl.

[93] El-Serag HB (2012). Epidemiology of viral hepatitis and hepatocellular carcinoma. Gastroenterology.

[94] Yang JD, Hainaut P, Gores GJ, Amadou A, Plymoth A, Roberts LR (2019). A global view of hepatocellular carcinoma: trends, risk, prevention and management. Nat Rev Gastroenterol Hepatol.

[95] Tarao K, Ohkawa S, Shimizu A, Harada M, Nakamura Y, Ito Y (1993). The male preponderance in incidence of hepatocellular carcinoma in cirrhotic patients may depend on the higher DNA synthetic activity of cirrhotic tissue in men. Cancer.

[96] Lui WY, Lin HL, Chau GY, Liu TY, Chi CW (2000). Male predominance in hepatocellular carcinoma: new insight and a possible therapeutic alternative. Med Hypotheses.

[97] Nagasue N, Kohno H (1992). Hepatocellular carcinoma and sex hormones. HPB Surg.

[98] Ruggieri A, Barbati C, Malorni W (2010). Cellular and molecular mechanisms involved in hepatocellular carcinoma gender disparity. Int J Cancer.

[99] Dhir RN, Dworakowski W, Thangavel C, Shapiro BH (2006). Sexually dimorphic regulation of hepatic isoforms of human cytochrome p450 by growth hormone. J Pharmacol Exp Ther.

[100] Park SJ, Jeong SY, Kim HJ (2006). Y chromosome loss and other genomic alterations in hepatocellular carcinoma cell lines analyzed by CGH and CGH array. Cancer Genet Cytogenet.

[101] Kido T, Lau YC (2016). Identification of a TSPY co-expression network associated with DNA hypomethylation and tumor gene expression in somatic cancers. J Genet Genomics.

[102] Kido T, Lau YC (2019). The Y-linked proto-oncogene TSPY contributes to poor prognosis of the male hepatocellular carcinoma patients by promoting the pro-oncogenic and suppressing the anti-oncogenic gene expression. Cell Biosci.

[103] Tsuei DJ, Hsu HC, Lee PH, Jeng YM, Pu YS, Chen CN (2004). RBMY, a male germ cell-specific RNA-binding protein, activated in human liver cancers and transforms rodent fibroblasts. Oncogene.

[104] Li S, Mo C, Huang S, Yang S, Lu Y, Peng Q (2014). Over-expressed Testis-specific Protein Y-encoded 1 as a novel biomarker for male hepatocellular carcinoma. PLoS ONE.

[105] Tsuei DJ, Lee PH, Peng HY, Lu HL, Su DS, Jeng YM (2011). Male germ cell-specific RNA binding protein RBMY: a new oncogene explaining male predominance in liver cancer. PLoS ONE.

[106] Chua HH, Tsuei DJ, Lee PH, Jeng YM, Lu J, Wu JF (2015). RBMY, a novel inhibitor of glycogen synthase kinase 3beta, increases tumor stemness and predicts poor prognosis of hepatocellular carcinoma. Hepatology.

[107] Kido T, Tabatabai ZL, Chen X, Lau YC (2020). Potential dual functional roles of the Y-linked RBMY in hepatocarcinogenesis. Cancer Sci.

[108] Yin YH, Li YY, Qiao H, Wang HC, Yang XA, Zhang HG (2005). TSPY is a cancer testis antigen expressed in human hepatocellular carcinoma. Br J Cancer.

[109] Salo P, Kaariainen H, Petrovic V, Peltomaki P, Page DC, de la Chapelle A (1995). Molecular mapping of the putative gonadoblastoma locus on the Y chromosome. Genes Chromosomes Cancer.

[110] Kido T, Lau YF (2008). The human Y-encoded testis-specific protein interacts functionally with eukaryotic translation elongation factor eEF1A, a putative oncoprotein. Int J Cancer.

[111] Li Y, Zhang DJ, Qiu Y, Kido T, Lau YC (2017). The Y-located proto-oncogene TSPY exacerbates and its X-homologue TSPX inhibits transactivation functions of androgen receptor and its constitutively active variants. Hum Mol Genet.

[112] Kido T, Lo RC, Li Y, Lee J, Tabatabai ZL, Ng IO (2014). The potential contributions of a Y-located protooncogene and its X homologue in sexual dimorphisms in hepatocellular carcinoma. Hum Pathol.

[113] Oram SW, Liu XX, Lee TL, Chan WY, Lau YF (2006). TSPY potentiates cell proliferation and tumorigenesis by promoting cell cycle progression in HeLa and NIH3T3 cells. BMC Cancer.

[114] Shirakawa H, Kuronuma T, Nishimura Y, Hasebe T, Nakano M, Gotohda N (2009). Glypican-3 is a useful diagnostic marker for a component of hepatocellular carcinoma in human liver cancer. Int J Oncol.

[115] Liu C, Ren YF, Dong J, Ke MY, Ma F, Monga SPS (2017). Activation of SRY accounts for male-specific hepatocarcinogenesis: implication in gender disparity of hepatocellular carcinoma. Cancer Lett.

[116] Kurabe N, Katagiri K, Komiya Y, Ito R, Sugiyama A, Kawasaki Y (2007). Deregulated expression of a novel component of TFTC/STAGA histone acetyltransferase complexes, rat SGF29, in hepatocellular carcinoma: possible implication for the oncogenic potential of c-Myc. Oncogene.

[117] Kurabe N, Murakami S, Tashiro F (2015). SGF29 and Sry pathway in hepatocarcinogenesis. World J Biol Chem.

[118] Brothman AR, Maxwell TM, Cui J, Deubler DA, Zhu XL (1999). Chromosomal clues to the development of prostate tumors. Prostate.

[119] Rawla P (2019). Epidemiology of prostate cancer. World J Oncol.

[120] Bray F, Ferlay J, Soerjomataram I, Siegel RL, Torre LA, Jemal A (2018). Global cancer statistics 2018: GLOBOCAN estimates of incidence and mortality worldwide for 36 cancers in 185 countries. CA Cancer J Clin.

[121] Yadav SK, Kumari A, Javed S, Ali S (2014). DYZ1 arrays show sequence variation between the monozygotic males. BMC Genet.

[122] Stahl PR, Kilgue A, Tennstedt P, Minner S, Krohn A, Simon R (2012). Y chromosome losses are exceedingly rare in prostate cancer and unrelated to patient age. Prostate.

[123] Perinchery G, Sasaki M, Angan A, Kumar V, Carroll P, Dahiya R (2000). Deletion of Y-chromosome specific genes in human prostate cancer. J Urol.

[124] Jangravi Z, Alikhani M, Arefnezhad B, Sharifi Tabar M, Taleahmad S, Karamzadeh R (2013). A fresh look at the male-specific region of the human Y chromosome. J Proteome Res.

[125] Vijayakumar S, Hall DC, Reveles XT, Troyer DA, Thompson IM, Garcia D (2006). Detection of recurrent copy number loss at Yp11.2 involving TSPY gene cluster in prostate cancer using array-based comparative genomic hybridization. Cancer Res.

[126] Nargesi MM, Ismail P, Razack AH, Pasalar P, Nazemi A, Oshkoor SA (2011). Linkage between prostate cancer occurrence and Y-chromosomal DYS loci in Malaysian subjects. Asian Pac J Cancer Prev.

[127] Eeles RA, Olama AA, Benlloch S, Saunders EJ, Leongamornlert DA, Tymrakiewicz M, et al. Identification of 23 new prostate cancer susceptibility loci using the iCOGS custom genotyping array. Nat Genet. 2013;45(4):385–91, 91e1–2. 10.1038/ng.2560 PMC3832790 23535732

[128] Han Y, Rand KA, Hazelett DJ, Ingles SA, Kittles RA, Strom SS (2016). Prostate cancer susceptibility in men of African Ancestry at 8q24. J Natl Cancer Inst.

[129] Yao L, Ren S, Zhang M, Du F, Zhu Y, Yu H (2015). Identification of specific DNA methylation sites on the Y-chromosome as biomarker in prostate cancer. Oncotarget.

[130] Lau YF, Zhang J (2000). Expression analysis of thirty one Y chromosome genes in human prostate cancer. Mol Carcinog.

[131] Dasari VK, Goharderakhshan RZ, Perinchery G, Li LC, Tanaka Y, Alonzo J (2001). Expression analysis of Y chromosome genes in human prostate cancer. J Urol.

[132] Khosravi P, Gazestani VH, Asgari Y, Law B, Sadeghi M, Goliaei B (2014). Network-based approach reveals Y chromosome influences prostate cancer susceptibility. Comput Biol Med.

[133] Leng X, Liu M, Tao D, Yang B, Zhang Y, He T (2021). Epigenetic modification-dependent androgen receptor occupancy facilitates the ectopic TSPY1 expression in prostate cancer cells. Cancer Sci.

[134] Lau YC, Li Y, Kido T (2019). Battle of the sexes: contrasting roles of testis-specific protein Y-encoded (TSPY) and TSPX in human oncogenesis. Asian J Androl.

[135] Li Y, Lau YF (2008). TSPY and its X-encoded homologue interact with cyclin B but exert contrasting functions on cyclin-dependent kinase 1 activities. Oncogene.

[136] Jangravi Z, Tabar MS, Mirzaei M, Parsamatin P, Vakilian H, Alikhani M (2015). Two splice variants of Y chromosome-located lysine-specific demethylase 5D have distinct function in prostate cancer cell line (DU-145). J Proteome Res.

[137] Jangravi Z, Najafi M, Shabani M (2016). Investigation of histone lysine-specific demethylase 5D KDM5D) isoform expression in prostate cancer cell lines: a system approach. Iran Biomed J.

[138] Komura K, Jeong SH, Hinohara K, Qu F, Wang X, Hiraki M (2016). Resistance to docetaxel in prostate cancer is associated with androgen receptor activation and loss of KDM5D expression. Proc Natl Acad Sci USA.

[139] Komura K, Yoshikawa Y, Shimamura T, Chakraborty G, Gerke TA, Hinohara K (2018). ATR inhibition controls aggressive prostate tumors deficient in Y-linked histone demethylase KDM5D. J Clin Invest.

[140] Li N, Dhar SS, Chen TY, Kan PY, Wei Y, Kim JH (2016). JARID1D is a suppressor and prognostic marker of prostate cancer invasion and metastasis. Cancer Res.

[141] Evans JR, Feng FY, Chinnaiyan AM (2016). The bright side of dark matter: lncRNAs in cancer. J Clin Invest.

[142] Xiao G, Yao J, Kong D, Ye C, Chen R, Li L (2019). The long noncoding RNA TTTY15, which is located on the Y chromosome, promotes prostate cancer progression by sponging let-7. Eur Urol.

[143] Batool A, Karimi N, Wu XN, Chen SR, Liu YX (2019). Testicular germ cell tumor: a comprehensive review. Cell Mol Life Sci.

[144] Segal R (2006). Surveillance programs for stage I nonseminomatous germ cell tumors of the testis. Urol Oncol.

[145] Singla N, Lafin JT, Ghandour RA, Kaffenberger S, Amatruda JF, Bagrodia A (2019). Genetics of testicular germ cell tumors. Curr Opin Urol.

[146] Moreno-Mendoza D, Casamonti E, Paoli D, Chianese C, Riera-Escamilla A, Giachini C (2019). gr/gr deletion predisposes to testicular germ cell tumour independently from altered spermatogenesis: results from the largest European study. Eur J Hum Genet.

[147] Linger R, Dudakia D, Huddart R, Easton D, Bishop DT, Stratton MR (2007). A physical analysis of the Y chromosome shows no additional deletions, other than Gr/Gr, associated with testicular germ cell tumour. Br J Cancer.

[148] Anderson PD, Lam MY, Poirier C, Bishop CE, Nadeau JH (2009). The role of the mouse y chromosome on susceptibility to testicular germ cell tumors. Cancer Res.

[149] Machiela MJ, Dagnall CL, Pathak A, Loud JT, Chanock SJ, Greene MH (2017). Mosaic chromosome Y loss and testicular germ cell tumor risk. J Hum Genet.

[150] Li Y, Tabatabai ZL, Lee TL, Hatakeyama S, Ohyama C, Chan WY (2007). The Y-encoded TSPY protein: a significant marker potentially plays a role in the pathogenesis of testicular germ cell tumors. Hum Pathol.

[151] Kersemaekers AM, Honecker F, Stoop H, Cools M, Molier M, Wolffenbuttel K (2005). Identification of germ cells at risk for neoplastic transformation in gonadoblastoma: an immunohistochemical study for OCT3/4 and TSPY. Hum Pathol.

[152] Forsberg LA, Rasi C, Malmqvist N, Davies H, Pasupulati S, Pakalapati G (2014). Mosaic loss of chromosome Y in peripheral blood is associated with shorter survival and higher risk of cancer. Nat Genet.

[153] Qin N, Li N, Wang C, Pu Z, Ma Z, Jin G (2019). Association of mosaic loss of chromosome Y with lung cancer risk and prognosis in a Chinese population. J Thorac Oncol.

[154] Willis-Owen SAG, Domingo-Sabugo C, Starren E, Liang L, Freidin MB, Arseneault M (2021). Y disruption, autosomal hypomethylation and poor male lung cancer survival. Sci Rep.

[155] Arseneault M, Monlong J, Vasudev NS, Laskar RS, Safisamghabadi M, Harnden P (2017). Loss of chromosome Y leads to down regulation of KDM5D and KDM6C epigenetic modifiers in clear cell renal cell carcinoma. Sci Rep.

[156] Shen X, Hu K, Cheng G, Xu L, Chen Z, Du P (2019). KDM5D inhibit epithelial–mesenchymal transition of gastric cancer through demethylation in the promoter of Cul4A in male. J Cell Biochem.

[157] Gu J, Chu K (2021). Increased Mars2 expression upon microRNA-4661-5p-mediated KDM5D downregulation is correlated with malignant degree of gastric cancer cells. Cell Biol Int.

[158] Cai L, Chen Q, Fang S, Lian M, Lian M, Cai M (2020). ETV4 promotes the progression of gastric cancer through regulating KDM5D. Eur Rev Med Pharmacol Sci.

[159] Noveski P, Madjunkova S, Sukarova Stefanovska E, Matevska Geshkovska N, Kuzmanovska M, Dimovski A (2016). Loss of Y chromosome in peripheral blood of colorectal and prostate cancer patients. PLoS ONE.

[160] Asim A, Agarwal S, Avasthi KK, Sureka S, Rastogi N, Dean DD (2020). Investigation of LOY in prostate, pancreatic, and colorectal cancers in males: a case-control study. Expert Rev Mol Diagn.

[161] Agahozo MC, Timmermans MA, Sleddens HF, Foekens R, Trapman-Jansen AM, Schröder CP (2020). Loss of Y-chromosome during male breast carcinogenesis. Cancers.

[162] Westra W, Rygiel A, Mostafavi N, De Wit G, Roes A, Moons L (2020). The Y-chromosome F haplogroup contributes to the development of Barrett’s esophagus-associated esophageal adenocarcinoma in a white male population. Dis Esophagus.

[163] Loeser H, Wölwer CB, Alakus H, Chon S-H, Zander T, Buettner R (2020). Y chromosome loss is a frequent event in Barrett’s adenocarcinoma and associated with poor outcome. Cancers.

[164] Minner S, Kilgué A, Stahl P, Weikert S, Rink M, Dahlem R (2010). Y chromosome loss is a frequent early event in urothelial bladder cancer. Pathology.

[165] Veiga LC, Bergamo NA, Reis PP, Kowalski LP, Rogatto SR (2012). Loss of Y-chromosome does not correlate with age at onset of head and neck carcinoma: a case-control study. Braz J Med Biol Res.

[166] Hollows R, Wei W, Cazier JB, Mehanna H, Parry G, Halford G (2019). Association between loss of Y chromosome and poor prognosis in male head and neck squamous cell carcinoma. Head Neck.

[167] Shahrabi S, Khodadi E, Saba F, Shahjahani M, Saki N (2018). Sex chromosome changes in leukemia: cytogenetics and molecular aspects. Hematology.

[168] Lai IL, Chang YS, Chan WL, Lee YT, Yen JC, Yang CA (2019). Male-specific long noncoding RNA TTTY15 inhibits non-small cell lung cancer proliferation and metastasis via TBX4. Int J Mol Sci.

[169] Brownmiller T, Juric JA, Ivey AD, Harvey BM, Westemeier ES, Winters MT (2020). Y Chromosome LncRNA are involved in radiation response of male non-small cell lung cancer cells. Cancer Res.

[170] Wu S, Zhang L, Deng J, Guo B, Li F, Wang Y (2020). A novel micropeptide encoded by Y-linked LINC00278 links cigarette smoking and ar signaling in male esophageal squamous cell carcinoma. Cancer Res.

[171] Alikhani M, Karamzadeh R, Rahimi P, Adib S, Baharvand H, Salekdeh GH (2020). Human proteome project and human pluripotent stem cells: odd bedfellows or a perfect match?. J Proteome Res.

